# Supplementary material for: Modulation of TTR gene expression in the eye using modified siRNAs
Source: Nucleic Acids Res. 2025 May 19;53(9):gkaf409. doi: 10.1093/nar/gkaf409 (PMC12086539; doi:10.1093/nar/gkaf409)
Supplement: gkaf409_Supplemental_File [file gkaf409_supplemental_file.pdf]

Supplementary Information

Modulation of *TTR* Gene Expression in the Eye using Modified Duplex RNAs

Jiaxin Hu[1*], Xin Gong[2*], Jayanta Kundu[3], Dhrubajyoti Datta[3], Martin Egli, [4] Muthiah Manoharan[3], V. Vinod Mootha*[2,5] David R. Corey*[1]

[1]UT Southwestern Medical Center, Departments of Pharmacology and Biochemistry, Dallas TX 75390, USA

[2]UT Southwestern Medical Center, Department of Ophthalmology, Dallas, TX 75235, USA

[3]Alnylam Pharmaceuticals, Cambridge, MA, 04142 USA

[4]Vanderbilt University, Department of Biochemistry, School of Medicine Nashville, TN 37232, USA.

[5]UT Southwestern Medical Center, Eugene McDermott Center for Human Growth and Development, Dallas, TX, 75235, USA.

*These authors contributed equally to the work

To whom correspondence should be addressed. V. Vinod Mootha, vinod.mootha@utsouthwestern.edu; David R. Corey, David.corey@utsouthwestern.edu

Table of Contents

Oligonucleotide synthesis scheme	**S-2**
Oligonucleotide sequences and characterization	**S-3**
T_m data and representative heating curves	**S-4**
HPLC based hydrophobicity assay	**S-5**
Representative T_m curves.	**S-6**
^{1}H, ^{13}C, and ^{31}P NMR spectra for the new compounds	**S-7**
HPLC profiles of oligonucleotides	**S-19**
Crystal structure of human serum albumin in complex with myristic acid	**S-25**

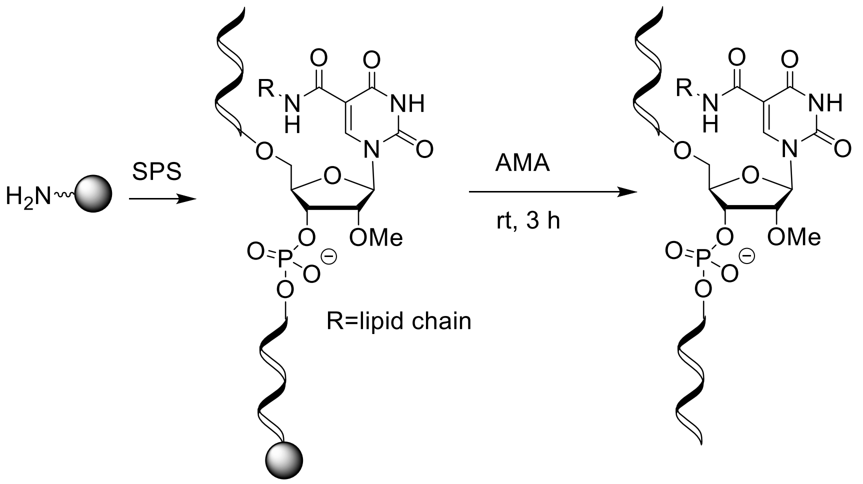

Figure S1: General synthesis scheme for oligonucleotides. SPS indicates solid-phase synthesis. AMA is (1:1) v/v 28-30 % aq. ammonia and 40 % aq. methylamine.

Table S1: Sequences and characterization of oligonucleotides used for this study.

Entry	Sequence of strands (5′-3′) [a]	Target	Mass(M-H)$^{-}$	
			Calcd.	Obsd.
ON1	c●a●uuu**I***A*a*UCC*ucacucua●a●a	SOD1	7101.03	7100.43
ON2	c●a●uuu**II***A*a*UCC*ucacucuaaaL8	SOD1	7558.50	7557.86
ON3	*A*●a●*C*a*G*u*G*u*UCU*u*G*c*U*c*U*a*U*a*A*L8	TTR	7093.48	7091.88
ON4	*A*●a●*C*a*G***I***G*u*UCU*u*G*c*U*c*U*a*U*a*A*L8	TTR	7360.94	7360.45
ON5	a●a●cagu*G*u*UCU*ugcucuauaaL8	ocTTR	7189.77	7188.92
ON6	a●a●cag**II***G*u*UCU*ugcucuauaaL8	ocTTR	7654.54	7653.82

[a]Chemical modifications are indicated as follows: ●, PS linkage; lower case, 2′-OMe; italicized upper case, 2′-F; L8, hydroxyprolinol-C6 linker.

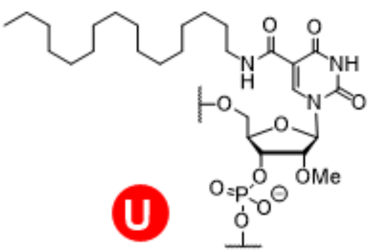

I

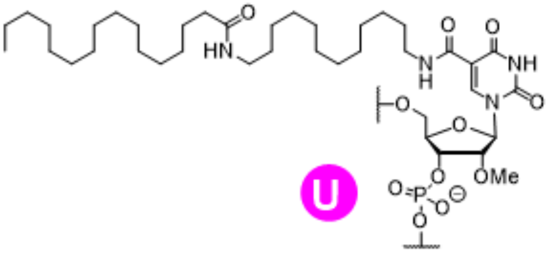

II

Table S2: Melting temperatures of duplexes

Entry	Bead diagram (sense at top from 5′ to 3′)[a]	Description	T_m[b](°C)	ΔT_m [c](°C)
1	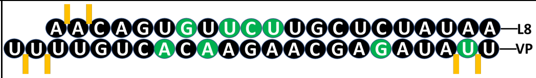	L8 control 2 with no lipid (TTRb)	63.8	NA
2	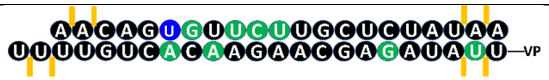	With 2′-O16 lipid (TTRb-C16)	62.3	-1.5
3	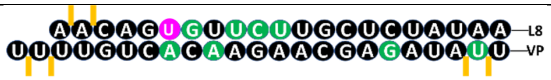	C5-functionalized-U containing C28 alkyl chain (TTRb-C28)	55.8	-8.0

[a]2′-fluoro (2′-F) and 2′-*O*-methyl (2′-OMe) nucleotides are indicated in green and black, respectively. Phosphorothioate (PS) linkages are indicated by orange lines. VP refers to vinyl phosphonate. [b]T_m values were obtained from the maxima of the first derivatives of the (average of three heating and three cooling) melting curves (*A*260 vs temperature) recorded in 0.1 × PBS buffer (pH 7.4) using 1.0 μM concentrations of each strand. [c]ΔT_m is the difference in melting temperature between the modified duplex and the reference duplex (Entry 1) which had a T_m of 63.8 °C. Cooling curves were recorded from 95 to 25 °C with a cooling rate of 1 °C/min. Heating curves were recorded from 25 to 95 °C with a heating rate of 1 °C/min. The T_m values are accurate within ±1.0 °C.

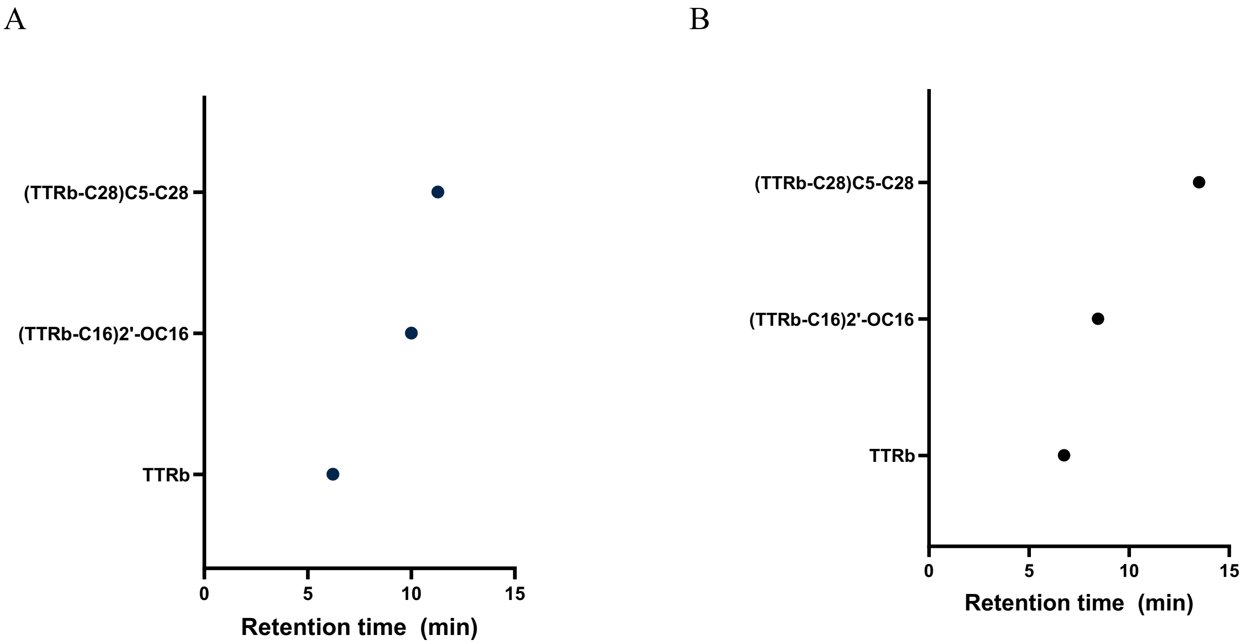

Figure S2. Plotted (RP-HPLC) retention time of A) ocTTR duplexes B) ocTTR single strands

A

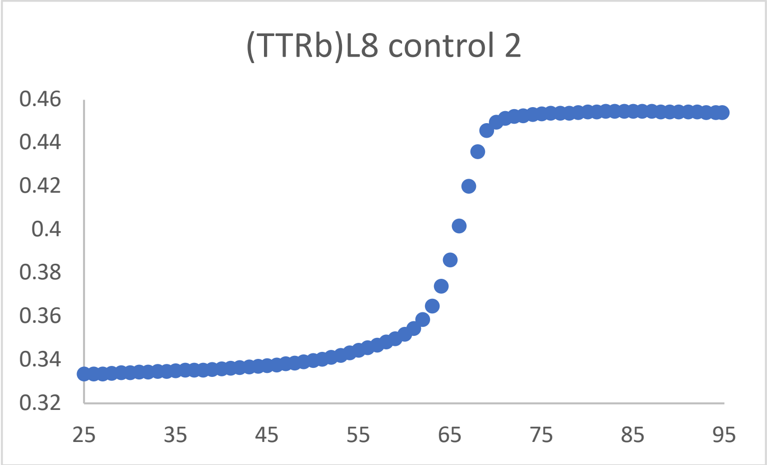

B

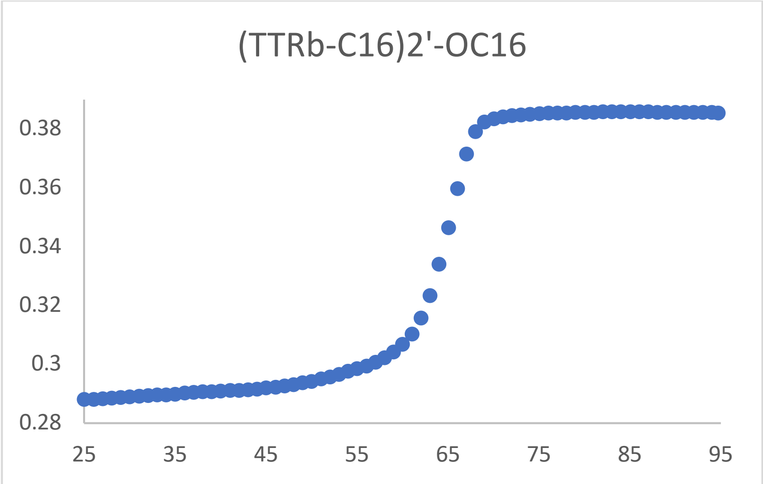

C

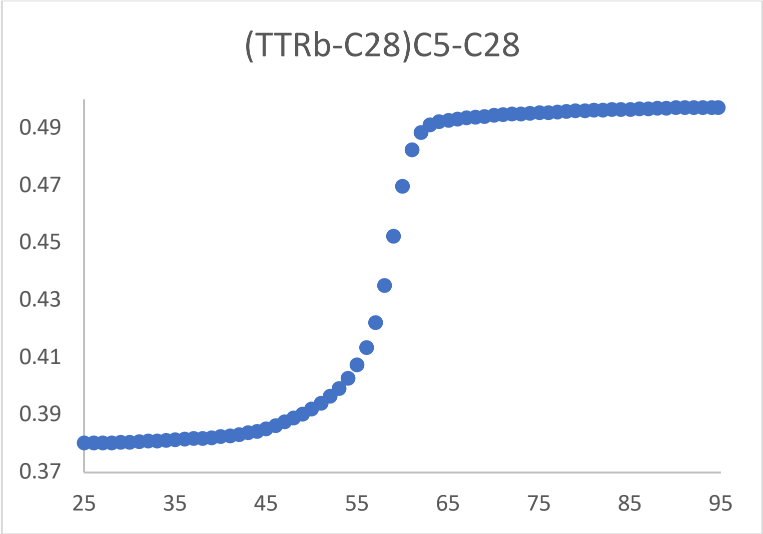

Figure S3: Representative heating curves of thermal melting study of A) TTRb B) (TTRb-C16)2′-OC16 and C) (TTRb-C28) C5-C28. Actual T_m in Table S2 is the average of three heating curves and three cooling curves.

Figure S4. Analytical data. NMR and HPLC.

^{1}H NMR of **1** (600 MHz) in $CDCl_3$

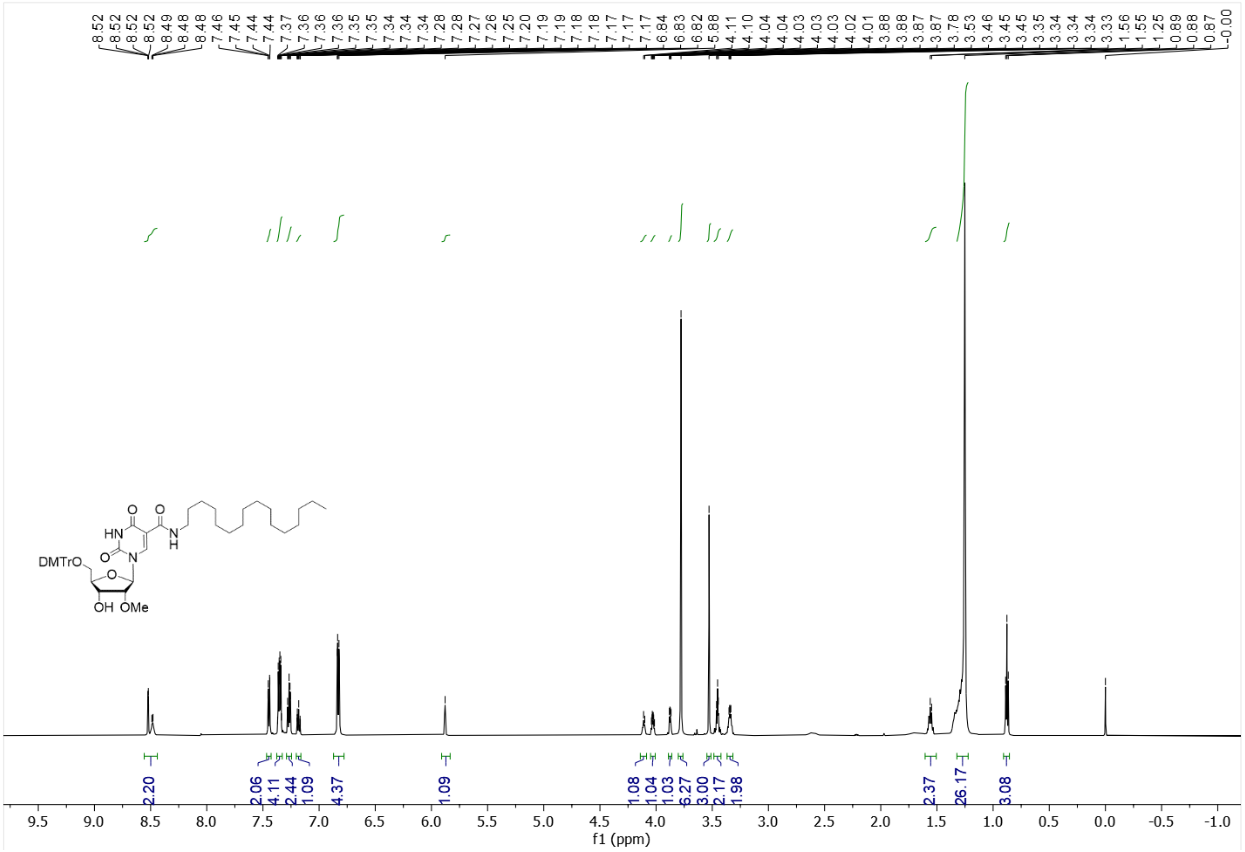

Figure S4. Analytical data. NMR and HPLC (cont.)

^{13}C NMR of **1** (151 MHz) in $CDCl_3$

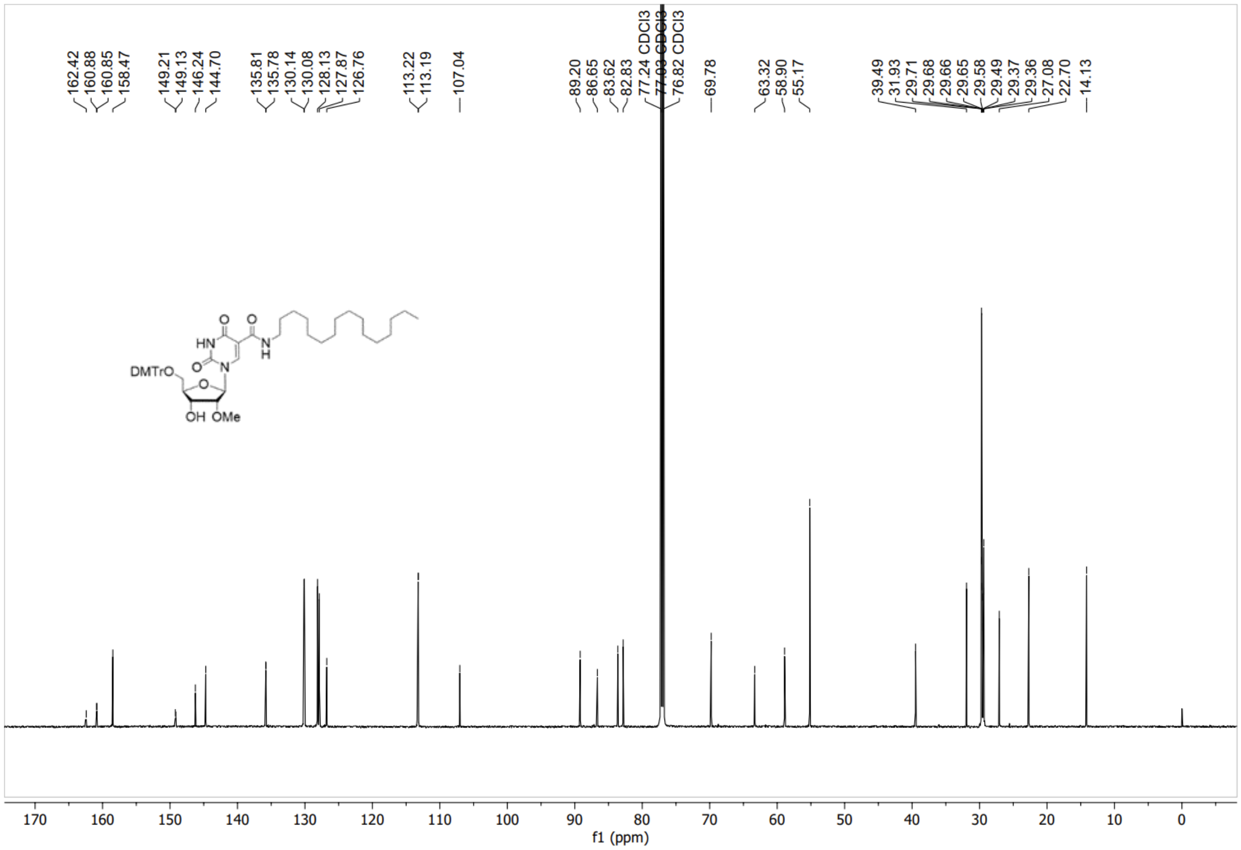

Figure S4. Analytical data. NMR and HPLC (cont.)

^{1}H NMR of **2** (600 MHz) in $CDCl_3$

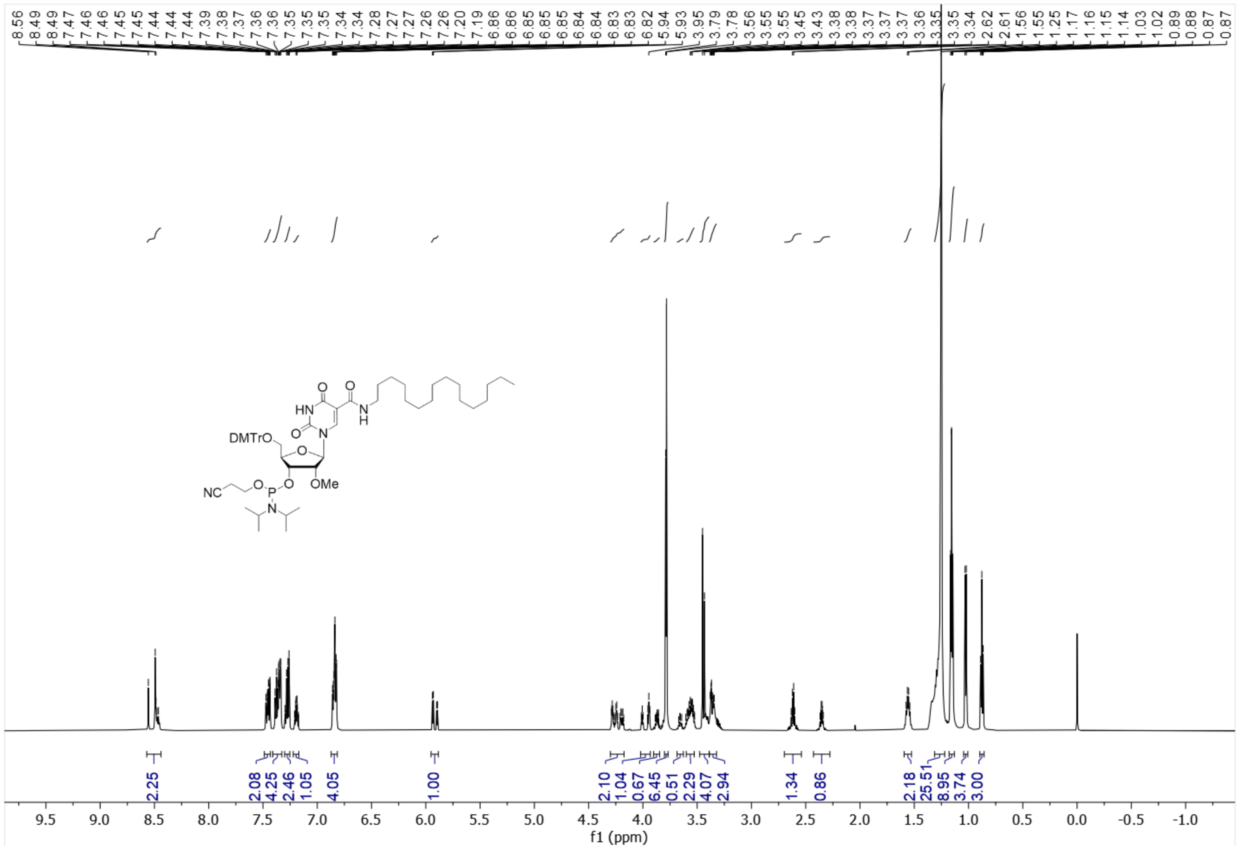

Figure S4. Analytical data. NMR and HPLC (cont.)

^{13}C NMR of **2** (151 MHz) in $CDCl_3$

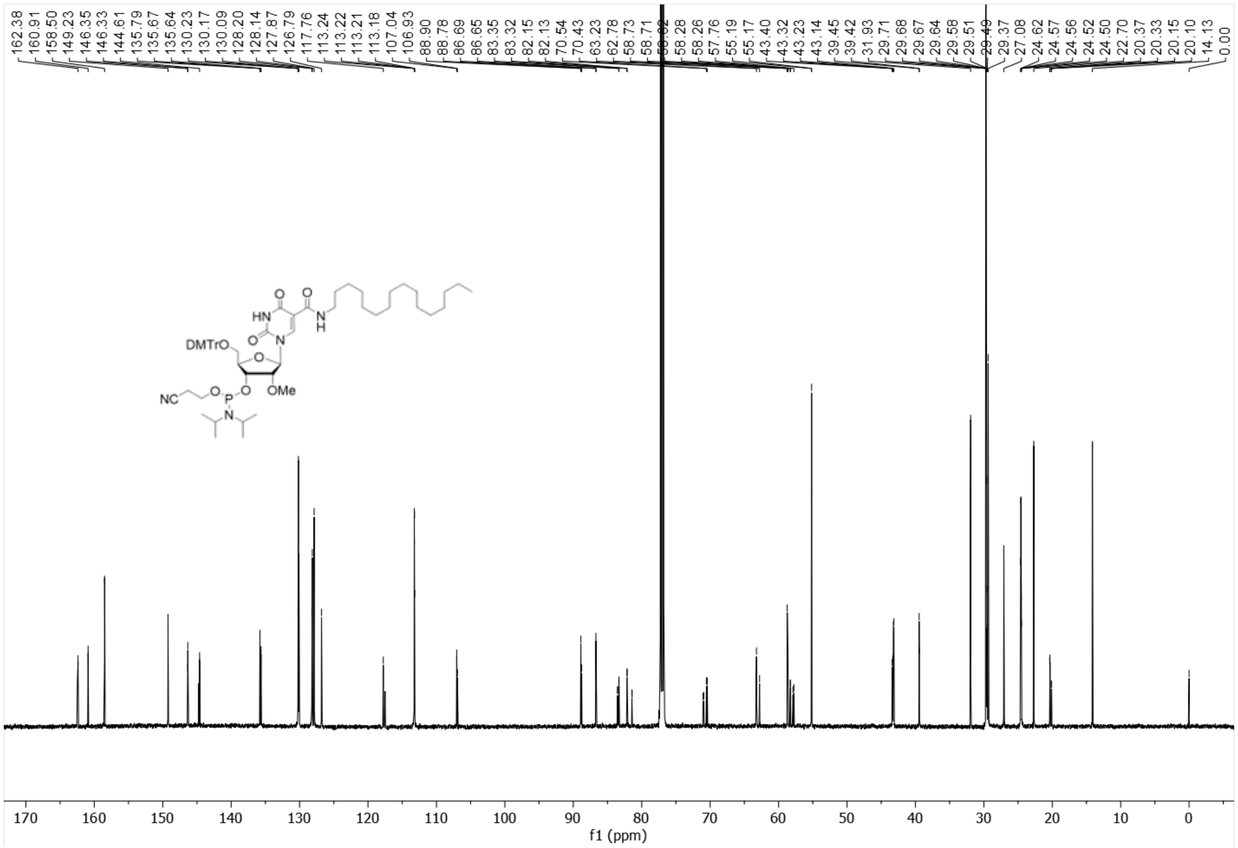

Figure S4. Analytical data. NMR and HPLC (cont.)

^{31}P NMR of **2** (243 MHz) in $CDCl_3$

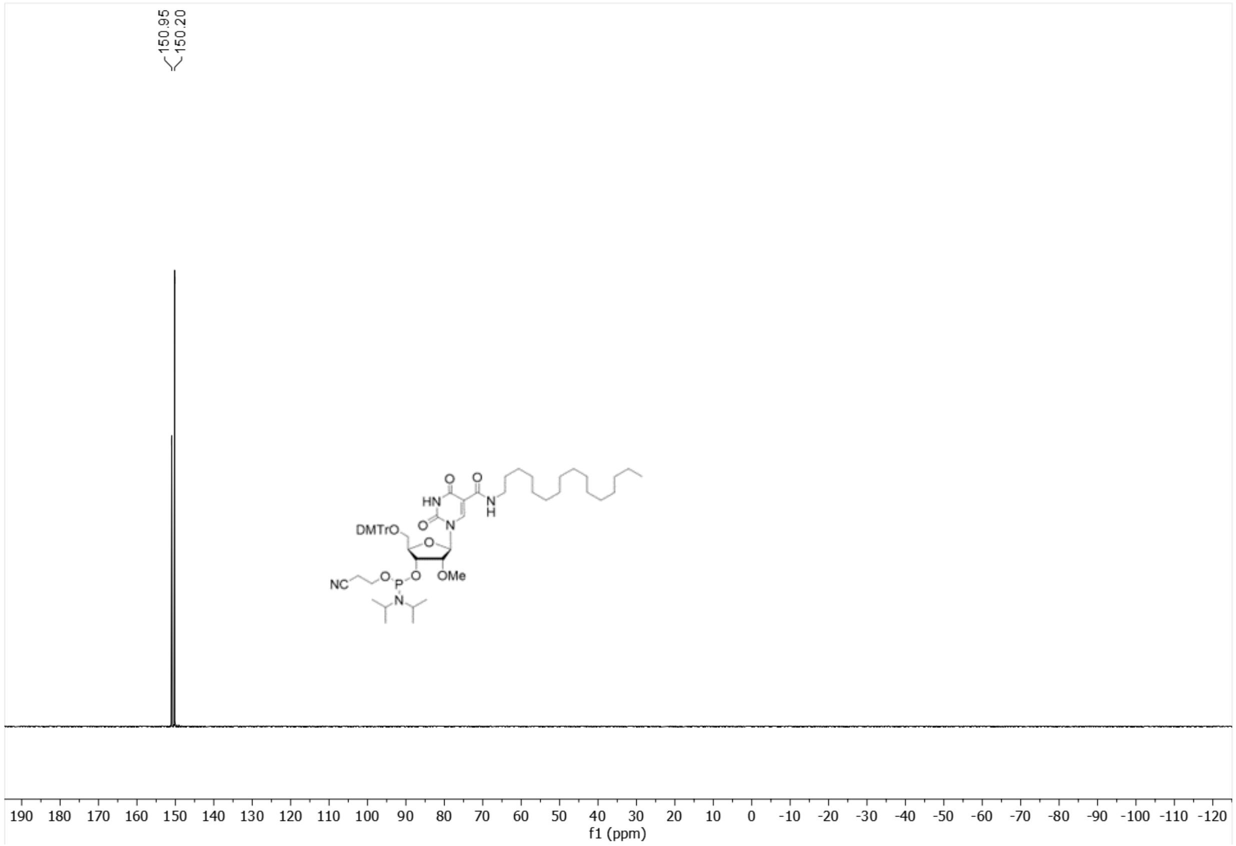

Figure S4. Analytical data. NMR and HPLC (cont.)

^{1}H NMR of **3** (600 MHz) in $CDCl_3$

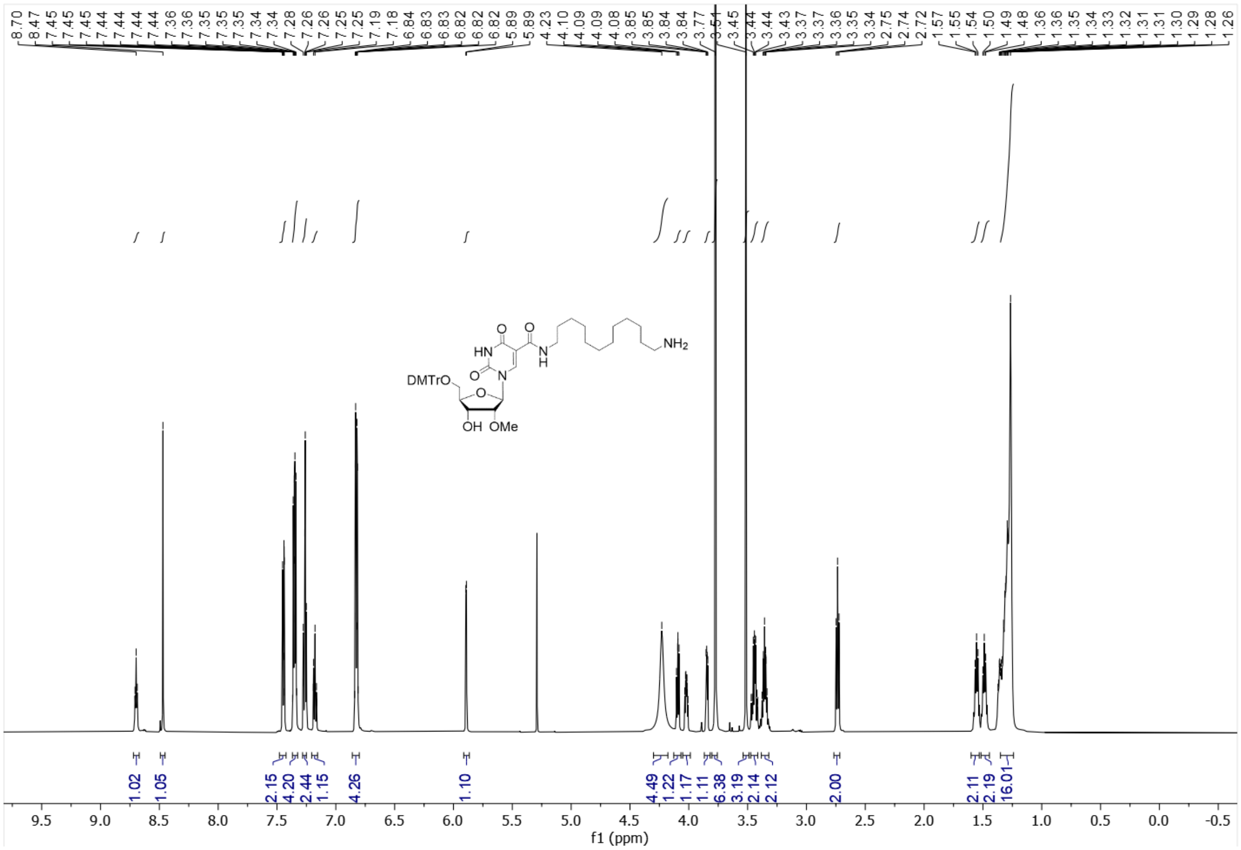

Figure S4. Analytical data. NMR and HPLC (cont.)

^{13}C NMR of **3** (151 MHz) in $CDCl_3$

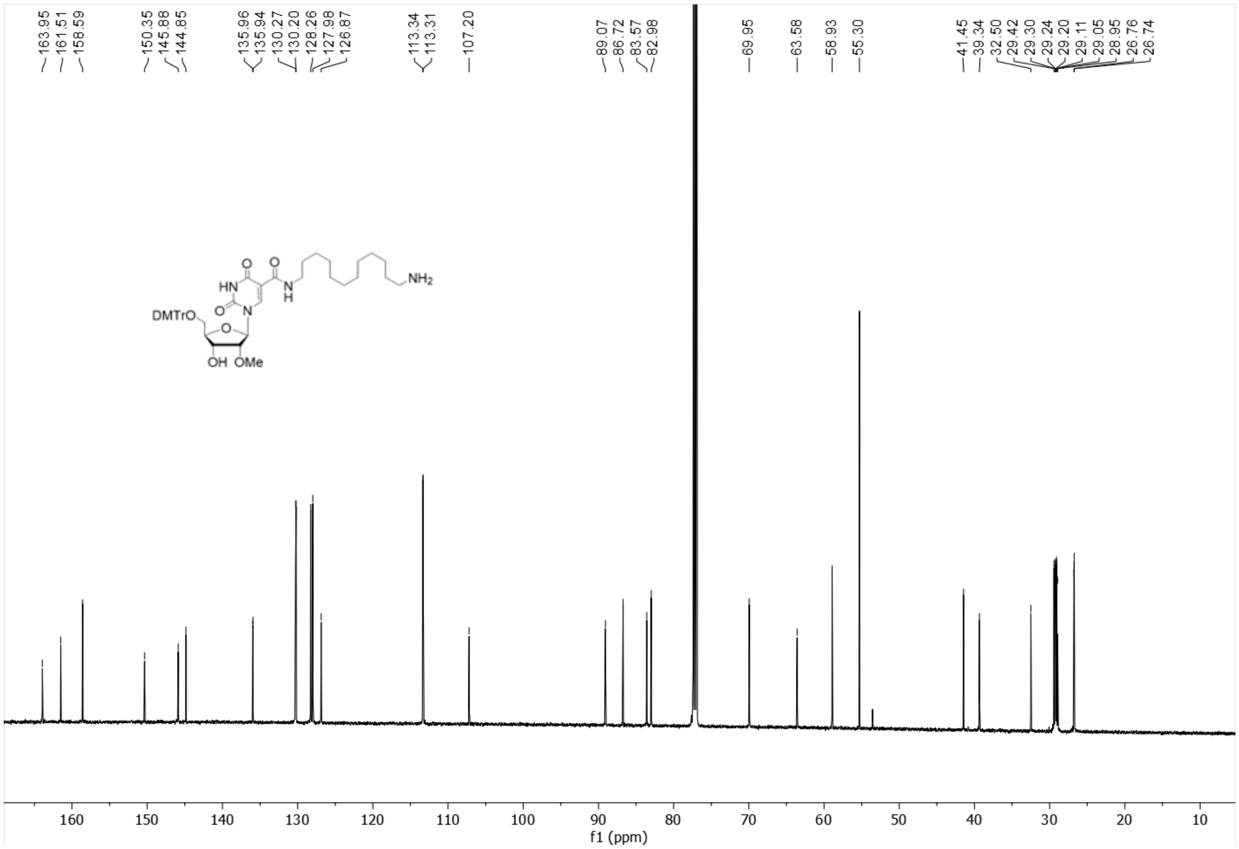

Figure S4. Analytical data. NMR and HPLC (cont.)

^{1}H NMR of **4**(600 MHz) in $CDCl_3$

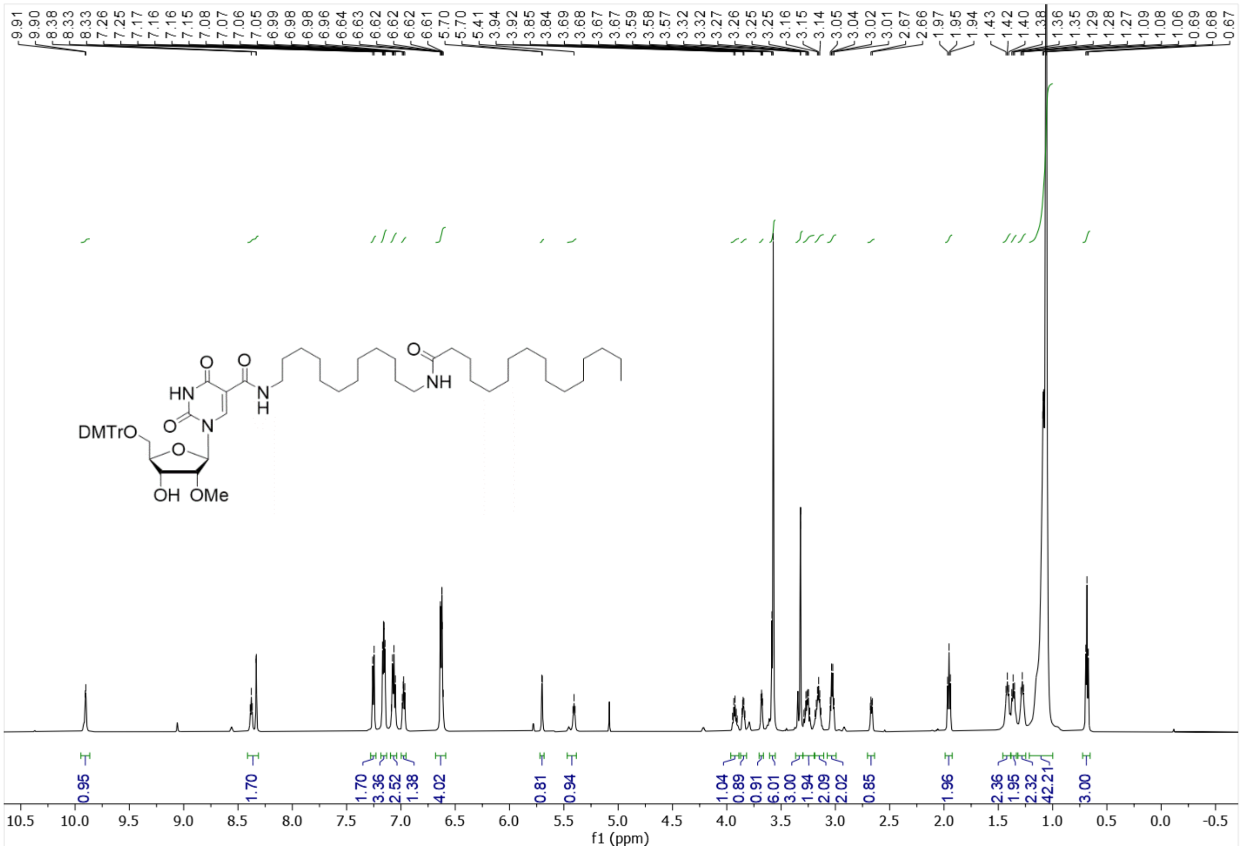

Figure S4. Analytical data. NMR and HPLC (cont.)

^{13}C NMR of **4** (151 MHz) in $CDCl_3$

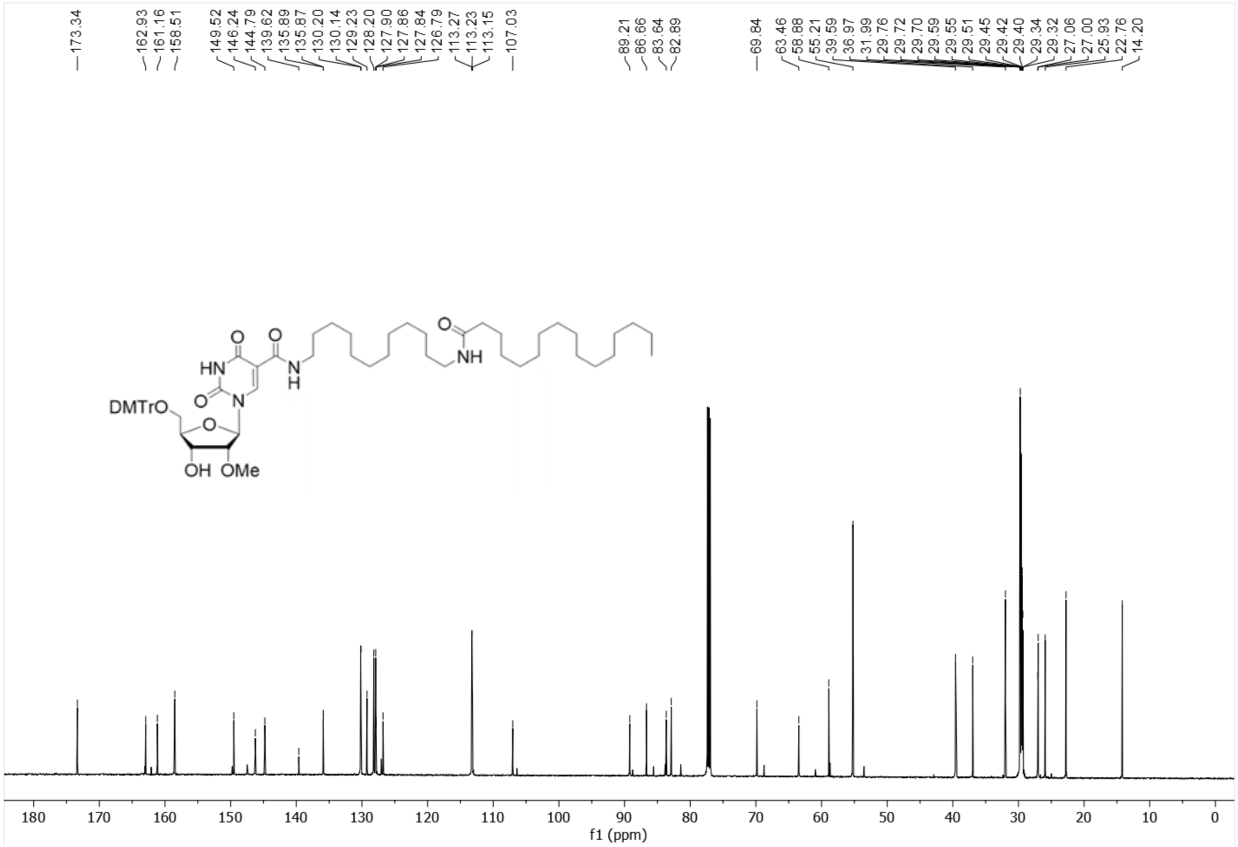

Figure S4. Analytical data. NMR and HPLC (cont.)

^{1}H NMR of **5** (600 MHz) in $CDCl_3$

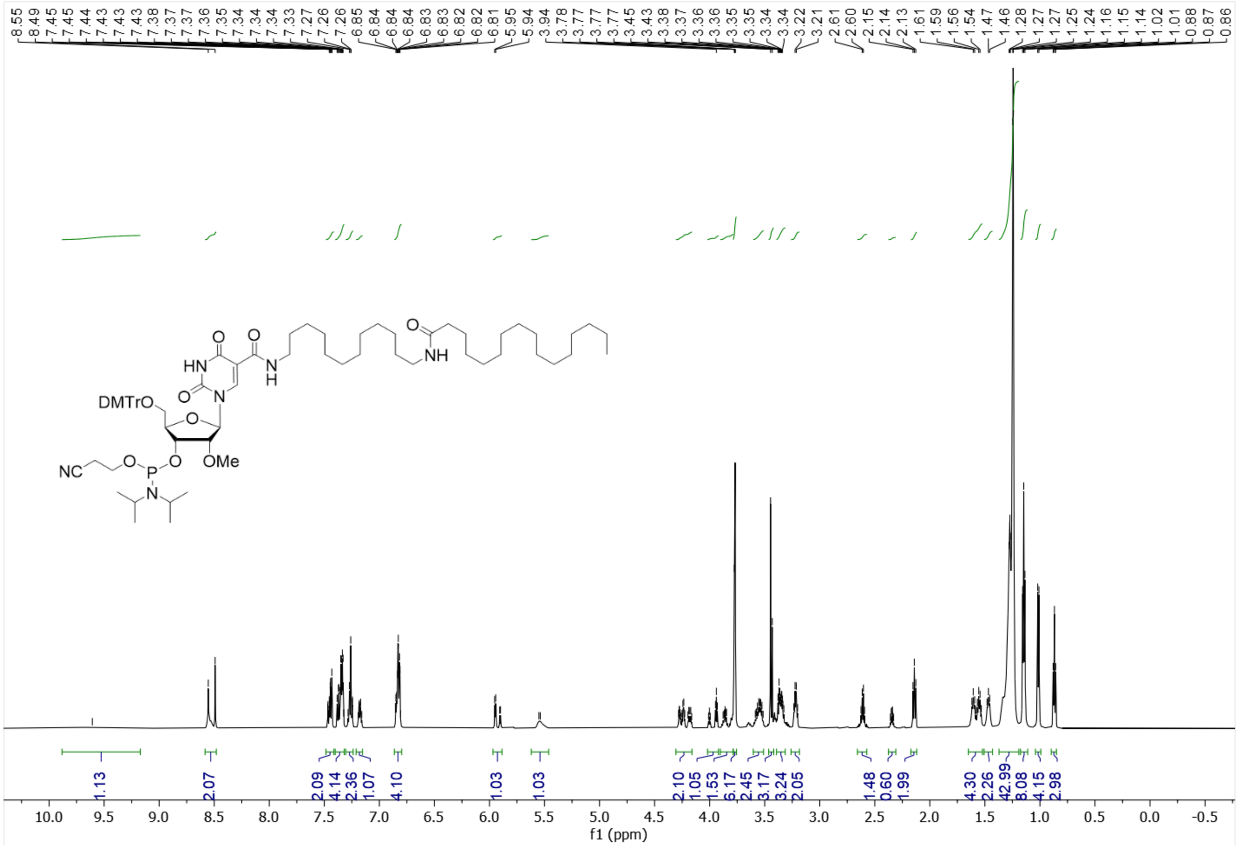

Figure S4. Analytical data. NMR and HPLC (cont.)

^{13}C NMR of **5** (151 MHz) in $CDCl_3$

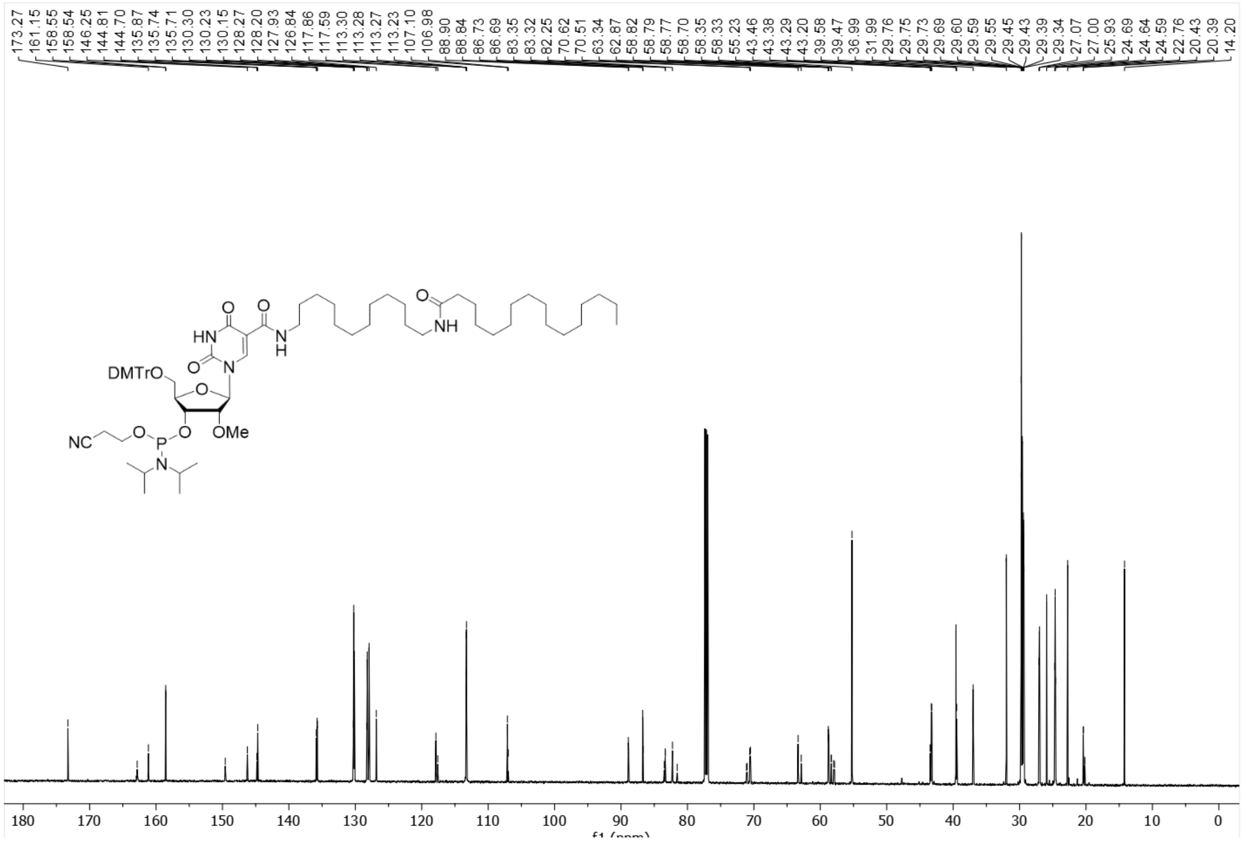

Figure S4. Analytical data. NMR and HPLC (cont.)

^{31}P NMR of **5** (243 MHz) in $CDCl_3$

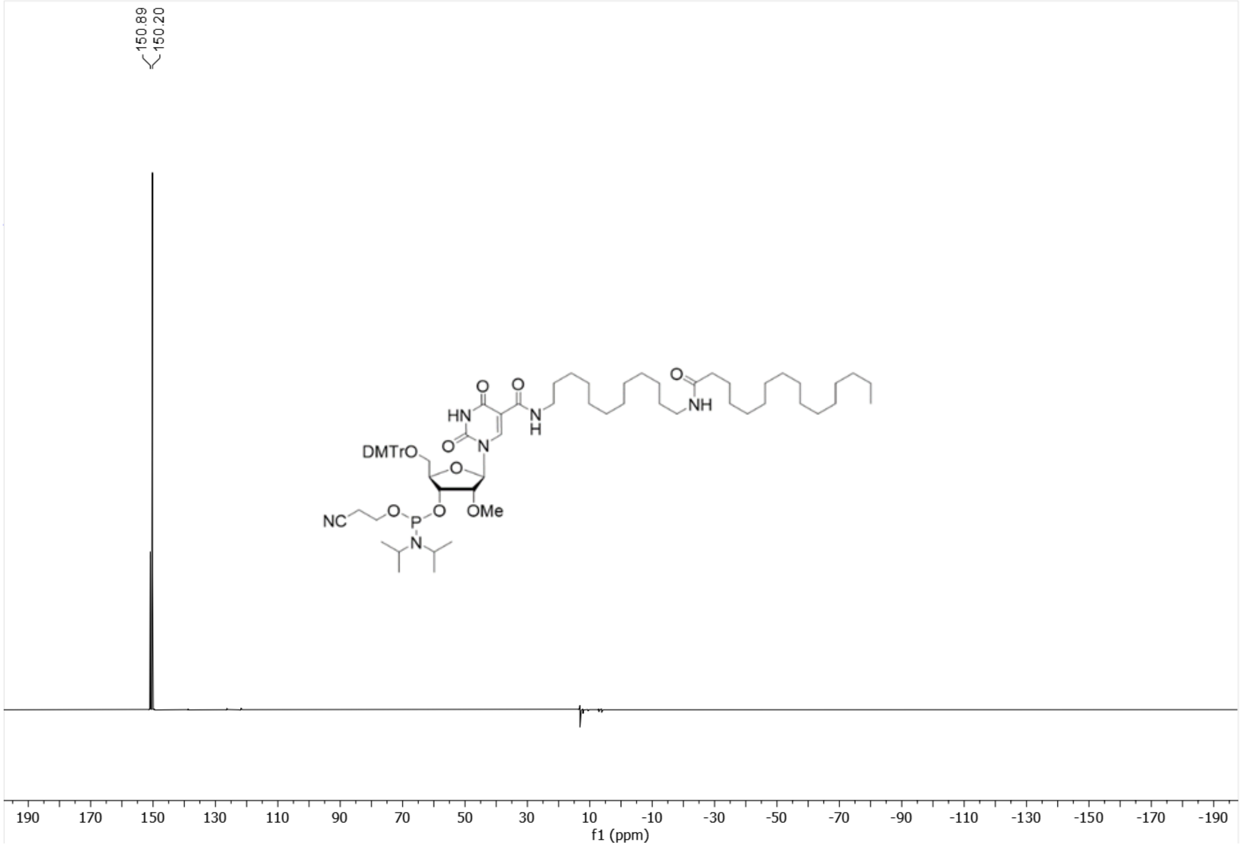

Figure S4. Analytical data. NMR and HPLC (cont.)

HPLC chromatograms of oligonucleotides, HPLC (Upper spectra is for UV signal and lower spectra is for mass signal)

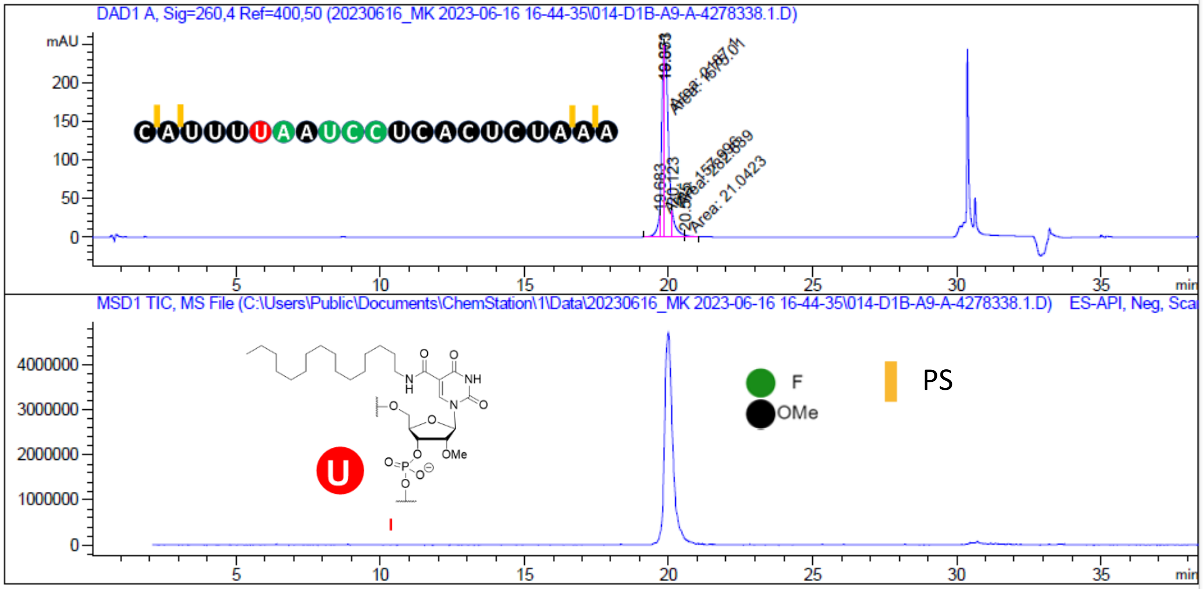


```
=====================================================================
                         Area Percent Report
=====================================================================

Sorted By             :      Signal
Multiplier            :      1.0000
Dilution              :      1.0000
Do not use Multiplier & Dilution Factor with ISTDs


Signal 1: DAD1 A, Sig=260,4 Ref=400,50
```


Peak #	RetTime [min]	Type	Width [min]	Area [mAU*s]	Height [mAU]	Area %
1	19.683	MF	0.0954	157.99622	27.60995	3.7406
2	19.861	MF	0.1053	1575.00513	249.18042	37.2890
3	19.873	MF	0.1452	2187.09937	250.99039	51.7806
4	20.123	MF	0.0986	282.63901	35.39711	6.6916
5	20.575	FM	0.1595	21.04233	2.19864	0.4982

Totals : 4223.78205 565.37650

Reverse-phase HPLC profile for **ON1**.

Figure S4. Analytical data. NMR and HPLC (cont.)

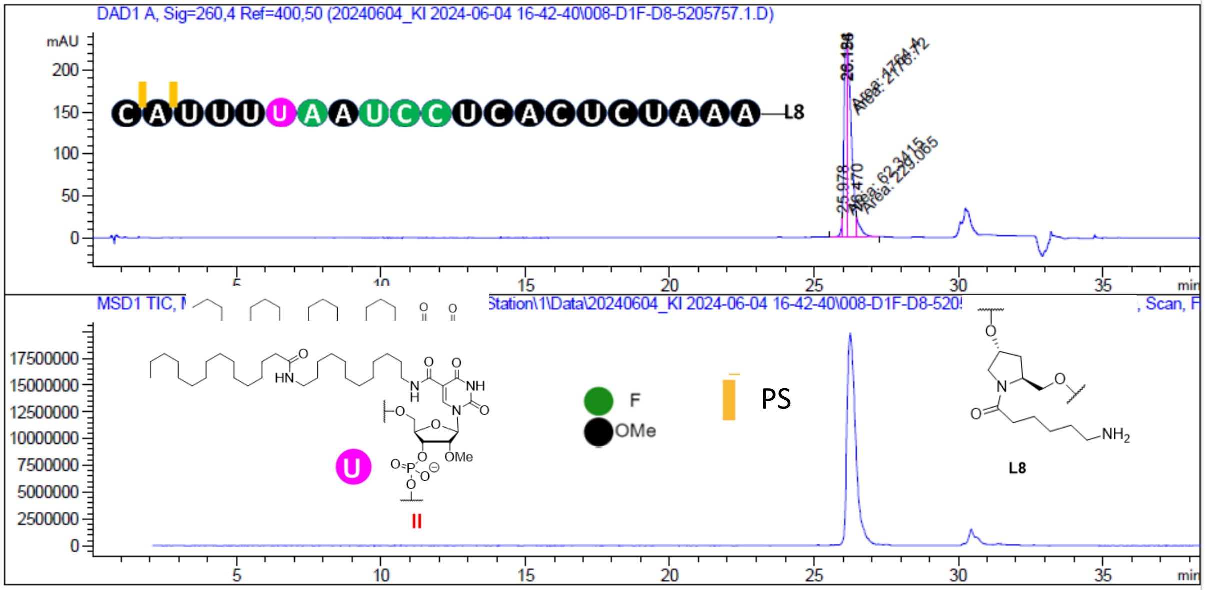

==

Area Percent Report

==

Sorted By : Signal
Multiplier : 1.0000
Dilution : 1.0000
Do not use Multiplier & Dilution Factor with ISTDs

Signal 1: DAD1 A, Sig=260,4 Ref=400,50

Data File C:\Users\P...ation\1\Data\20240604_KI 2024-06-04 16-42-40\008-D1F-D8-5205757.1.D
Sample Name: 5205757.1

Peak #	RetTime [min]	Type	Width [min]	Area [mAU*s]	Height [mAU]	Area %
1	25.978	MF	0.0448	62.34154	23.21166	1.4729
2	26.124	MF	0.1273	1764.40369	230.97623	41.6868
3	26.156	FM	0.1607	2176.71704	225.81992	51.4283
4	26.470	FM	0.1534	229.06462	24.89195	5.4120

Totals : 4232.52689 504.89976

Figure S4. Analytical data. NMR and HPLC (cont.)

Reverse-phase HPLC profile for **ON2**.

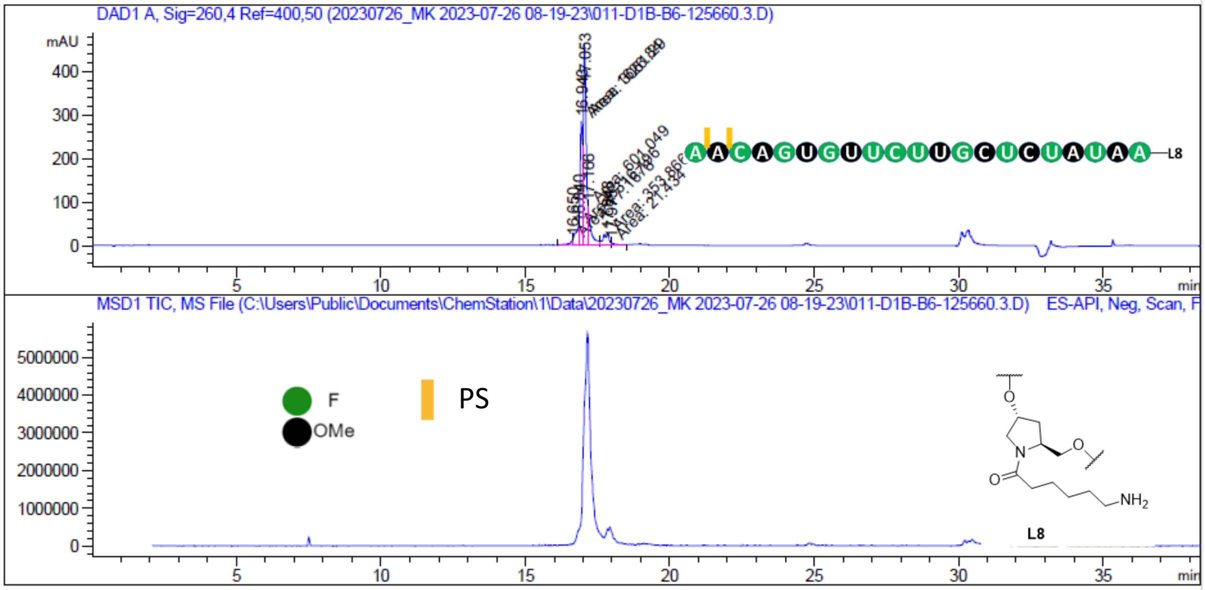

==

Area Percent Report

==

Sorted By : Signal
Multiplier : 1.0000
Dilution : 1.0000
Do not use Multiplier & Dilution Factor with ISTDs

Signal 1: DAD1 A, Sig=260,4 Ref=400,50

1290 Infinity2 LCMS 1 7/26/2023 4:02:26 PM SYSTEM Page 1 of 2

Data File C:\Users\P...tation\1\Data\20230726_MK 2023-07-26 08-19-23\011-D1B-B6-125660.3.D
Sample Name: 125660.3

Peak #	RetTime [min]	Type	Width [min]	Area [mAU*s]	Height [mAU]	Area %
1	16.650	MF	0.1044	77.16778	12.31973	1.2738
2	16.840	FM	0.1273	316.19632	41.40050	5.2196
3	16.943	MF	0.0954	1626.83618	284.21198	26.8550
4	17.053	MF	0.1102	3061.29224	462.98215	50.5344
5	17.166	MF	0.0941	601.04877	89.65131	9.9218
6	17.848	MF	0.2279	353.86646	25.87759	5.8415
7	17.975	FM	0.0890	21.43404	4.01590	0.3538
Totals :				6057.84178	920.45916	

Figure S4. Analytical data. NMR and HPLC (cont.)

Reverse-phase HPLC profile for **ON3**.

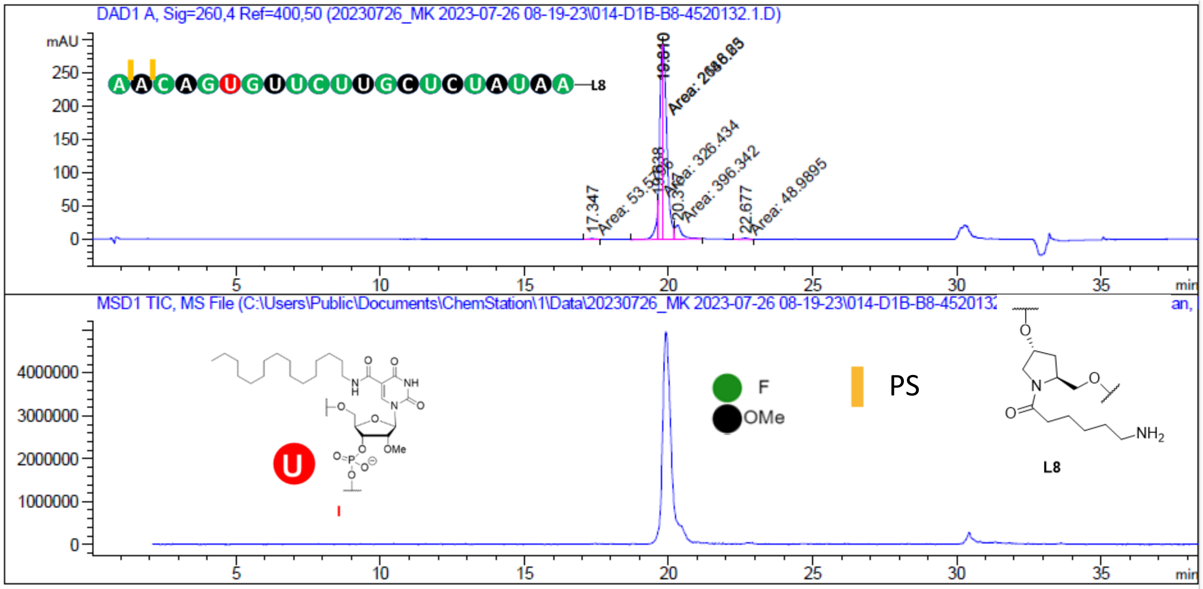

==

Area Percent Report

==

Sorted By : Signal
Multiplier : 1.0000
Dilution : 1.0000
Do not use Multiplier & Dilution Factor with ISTDs

Signal 1: DAD1 A, Sig=260,4 Ref=400,50

Peak #	RetTime [min]	Type	Width [min]	Area [mAU*s]	Height [mAU]	Area %
1	17.347	MM	0.4097	53.57963	2.17980	0.9466
2	19.638	MF	0.0916	326.43375	59.39338	5.7671
3	19.810	FM	0.1211	2148.62939	295.61874	37.9601
4	19.810	FM	0.1514	2686.25342	295.63287	47.4584
5	20.317	FM	0.3142	396.34195	21.02293	7.0022

1290 Infinity2 LCMS 1 7/27/2023 12:43:28 PM SYSTEM Page 1 of 2

Data File C:\Users\P...ation\1\Data\20230726_MK 2023-07-26 08-19-23\014-D1B-B8-4520132.1.D
Sample Name: 4520132.1

Peak #	RetTime [min]	Type	Width [min]	Area [mAU*s]	Height [mAU]	Area %
6	22.677	MM	0.3396	48.98952	2.40406	0.8655

Totals : 5660.22766 676.25179

Figure S4. Analytical data. NMR and HPLC (cont.)

Reverse-phase HPLC profile for **ON4**.

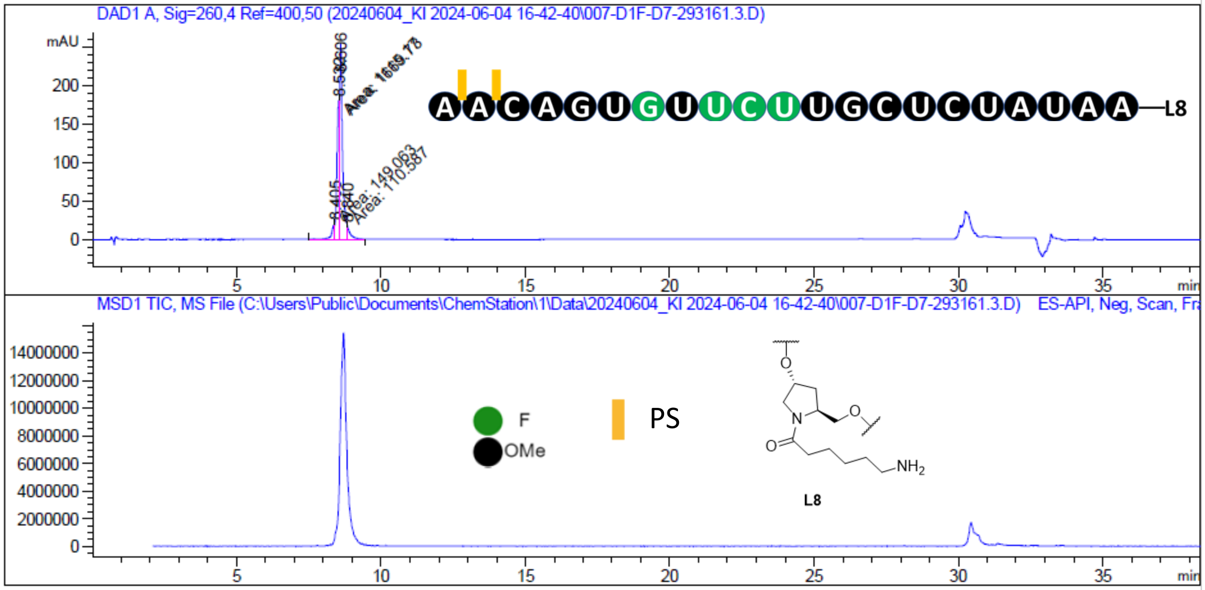


```
======================================================================
                          Area Percent Report
======================================================================

Sorted By             :      Signal
Multiplier            :      1.0000
Dilution              :      1.0000
Do not use Multiplier & Dilution Factor with ISTDs


Signal 1: DAD1 A, Sig=260,4 Ref=400,50
```


Peak #	RetTime [min]	Type	Width [min]	Area [mAU*s]	Height [mAU]	Area %
1	8.405	MF	0.1256	149.06302	19.78739	4.8961
2	8.532	MF	0.1031	1115.17236	180.19226	36.6285
3	8.606	FM	0.1092	1669.73010	254.80200	54.8432
4	8.840	FM	0.1108	110.58685	16.62820	3.6323

Totals : 3044.55233 471.40985

Figure S4. Analytical data. NMR and HPLC (cont.)

Reverse-phase HPLC profile for **ON5**.

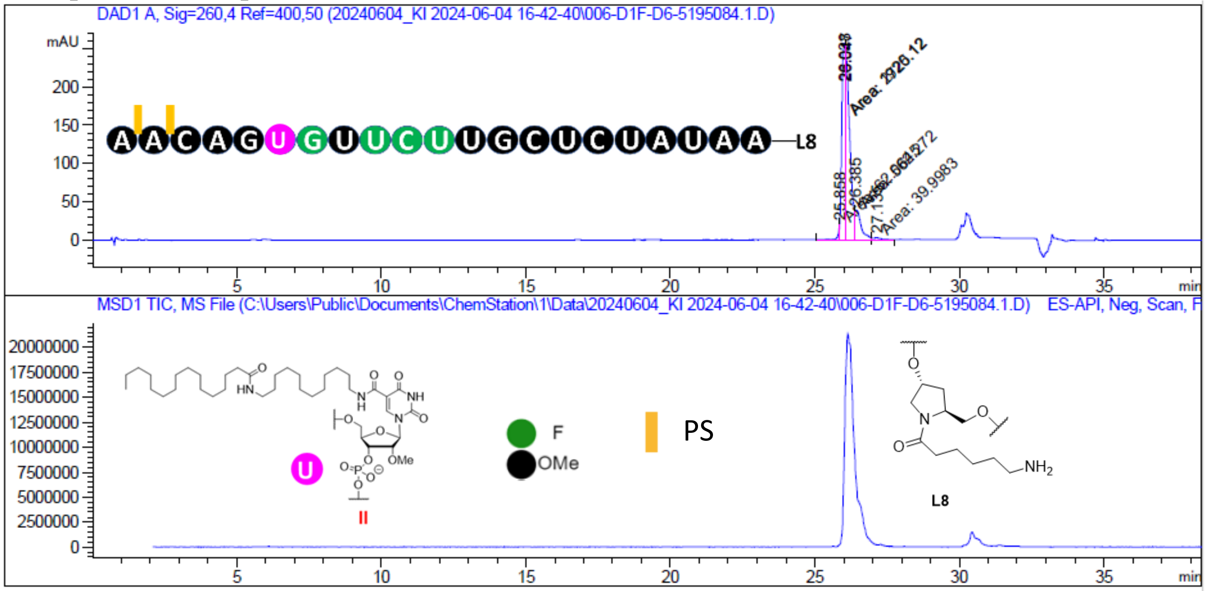


```
=====================================================================
                         Area Percent Report
=====================================================================

Sorted By              :      Signal
Multiplier             :      1.0000
Dilution               :      1.0000
Do not use Multiplier & Dilution Factor with ISTDs


Signal 1: DAD1 A, Sig=260,4 Ref=400,50
```


Data File C:\Users\P...ation\1\Data\20240604_KI 2024-06-04 16-42-40\006-D1F-D6-5195084.1.D
Sample Name: 5195084.1

Peak #	RetTime [min]	Type	Width [min]	Area [mAU*s]	Height [mAU]	Area %
1	25.858	MF	0.0517	62.06452	20.01926	1.1689
2	26.028	MF	0.1251	1920.12292	255.85619	36.1634
3	26.047	FM	0.1789	2725.11841	253.92242	51.3246
4	26.385	MF	0.1779	562.27197	38.16459	10.5898
5	27.135	FM	0.2719	39.99833	2.45222	0.7533
Totals :				5309.57616	570.41468	

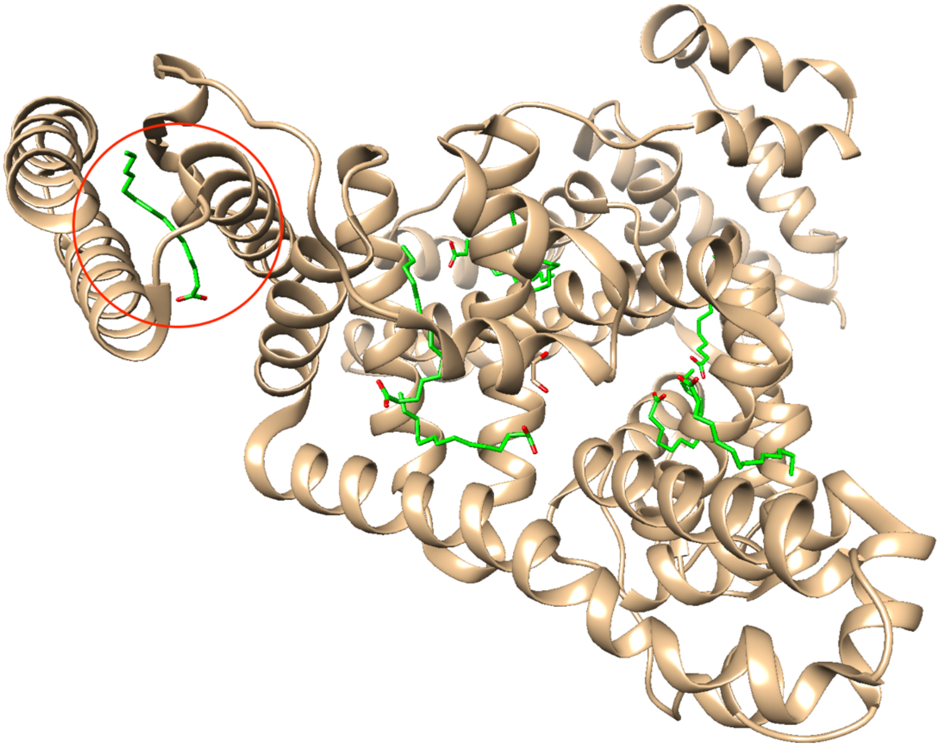

Figure S5. Crystal structure of human serum albumin in complex with myristic acid (PDB ID 8RCP). The protein is shown in cartoon mode and colored in tan and carbon atoms of eight bound fatty acid molecules are colored in green. We picked the myristic acid molecule marked with a red circle for building the TTRb-O2'-C16 and TTRb-C5-C28 siRNA duplexes bound to albumin.
